# Protective effects of licorice extract on ovarian morphology, oocyte maturation, and embryo development in PCOS-induced mice: An experimental study

**DOI:** 10.18502/ijrm.v13i10.7771

**Published:** 2020-10-13

**Authors:** Maryam Shamsi, Vahid Nejati, Gholamreza Najafi, Sana Khajeh Pour

**Affiliations:** ^1^Department of Histology and Embryology, Faculty of Science, Urmia University, Urmia, Iran.; ^2^Department of Anatomy, Faculty of Veterinary Medicine, Urmia University, Urmia, Iran.; ^3^Department of Biomedical and Pharmaceutical Sciences, Faculty of Pharmacy, Idaho State University, Pocatello, Idaho, USA.

**Keywords:** Polycystic ovary syndrome, Mice, Licorice, Histology, In vitro maturation, In vitro fertilization.

## Abstract

**Background:**

Polycystic ovary syndrome (PCOS) is an oxidative state resulting in ovarian dysfunction. Licorice is one of the natural antioxidants used for the treatment of infertility.

**Objective:**

To evaluate the effect of licorice extract on ovarian morphology, oocyte maturation, and embryo development in PCOS-induced mice.

**Materials and Methods:**

thirty-two female NMIR mice were divided into four groups (n = 8/each): control group receiving no treatment (group I); PCOS group injected with estradiol valerate once daily for 21 days (group II); and experimental groups receiving either 100 mg/kg (group III) or 150 mg/kg (group IV) licorice by gavage along with estradiol valerate once daily for 3 wk. Serum levels of the testosterone and estrogen were measured using ELISA kit. Histological study of ovaries was evaluated, and oocyte maturation, fertilization rate, and embryo development were determined after in vitro maturation.

**Results:**

Experimental groups (III, IV) had significantly higher testosterone and estradiol levels compared to the PCOS group (p ≤ 0.001). A significant increase in the number of healthy follicles (primary, preantral follicles) (p = 0.001), corpus luteum (p = 0.001) with significant decrease in the number of atretic follicles (primary, preantral, cystic follicles) (p ≤ 0.001) was seen in the experimental groups. Increase in the fertilization rate (p ≤ 0.001) and blastocyst stage embryos (p = 0.02, p = 0.004) were observed in the experimental groups.

**Conclusion:**

It appears that the two doses (100 mg and 150 mg) of licorice could decrease ovarian cyst and improve the fertilization rate of oocyte and embryo development in PCOS mice. However, there was no statistically significant difference between the two experimental groups.

## 1. Introduction

Polycystic ovary syndrome (PCOS) is a hormonal imbalance and metabolic disorder affecting about 8-13% of all women during their reproductive age. Anovulation, hyperandrogenism, polycystic ovary, hyperinsulinism, hirsutism, and elevated concentrations of LH are some of the major implications of PCOS (1, 2). It is seen that the increased enzymatic activities of 17αhydroxylase/17, 20 lyase, 3β-hydroxysteroid dehydrogenase, as well as the side chain cleavage enzyme lead to hyperandrogenism in patients with PCOS (3, 4). In addition, the biosynthesis of estrogens from androgens is catalyzed by aromatase; hence, a deficiency in aromatase activity may lead to intraovarian disturbances and hormonal imbalance (5).

An ovulatory cycle may lead to infertility resulting in a need for assisted reproductive techniques (6). Moreover, in vitro maturation (IVM) is a relatively new method in assisted reproductive techniques, in which immature oocytes are extracted from the ovaries without gonadotropin stimulation, thus limiting the risk of ovarian hyperstimulation syndrome compared to in vitro fertilization (IVF) technique (7). Currently, using various herbal remedies as natural antioxidants is suggested for improving all clinical features of PCOS to recover the menstrual cycle and normal serum hormones levels. Therefore, antioxidant supplementation are known to be effective in reducing testosterone, polycystic ovaries, and improving reproductive cycles (8, 9).

licorice (*Glycyrrhiza glabra*) has been used to treat different diseases for many years (10). Multiple biological activities such as the powerful antioxidative, antifatigue, antibacterial, antiviral, antiproliferative, and estrogenic activity of licorice have been demonstrated in several experimental evidence (11, 12). Therefore, some studies have suggested licorice extract as a possible candidate for the treatment of infertility as well as PCOS (13). Previous studies have revealed that blocking the activity of 3β-hydroxysteroid dehydrogenase (3HSD), 17-hydroxysteroid dehydrogenase (17HSD), and 17-20 lyase, and stimulating the activity of aromatase, while also affecting the 5a- and 5b-reductase activity, are some effects of licorice extract (14, 15). All these enzymes are involved in the synthesis and/or metabolism of androgens and estrogens (14). Lakshmi and colleagues demonstrated that inhibiting 17HSD, licorice could decrease serum testosterone in PCOS women, indicating that licorice has an anti-testosterone activity that may be beneficial in treating women with PCOS (16). Additionally, licorice exhibits estrogenic properties that could result in aromatase stimulation activity (17), and currently supplements of licorice are used for overweight PCOS patients (18). It was recently demonstrated that licorice extract can result in an improvement in IVF results in mice (19). Also, data show an improvement of PCOS symptoms such as imbalance in the hormonal levels and irregular ovarian follicles in letrozole-induced female rats (20).

To the best of our knowledge, possible beneficial and adverse effects of licorice extract along with IVM on female fertility have not been studied yet. As a result, the current study intended to examine the various concentrations of licorice extract on the histological changes of the ovaries along with oocyte maturation and embryo development in mice induced with PCOS.

## 2. Materials and Methods

### Chemicals

Material and reagents used in the present study were purchased from Sigma-Aldrich (Germany), unless otherwise mentioned.

### Animals and experimental design

Adult Female (6-7 wk old) and male (8-12 wk old) Naval Medical Research Institute (NMRI) mice were obtained from the breeding center at the Faculty of Science, Urmia University (Urmia, Iran). The mice were housed in a 12-hr light/dark cycle, at 25 ± 1°C and 50-60% humidity, and had free access to standard pellet diet and water ad libitum.

**Figure 1:**
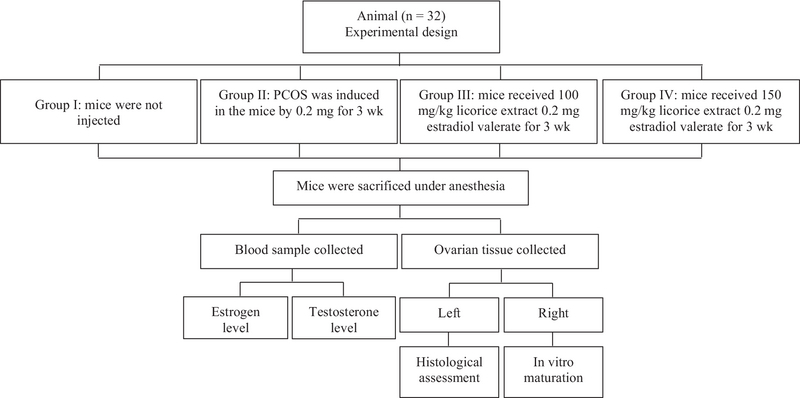
Flow chart of the experimental design.

The female mice were randomly assigned into four groups (n = 8/group) as follows:

• Group I: mice receiving no treatment (control group)

• Group II: PCOS was induced in mice through 0.2 mg estradiol valerate intramuscularly once daily for 3 wk.

• Group III: mice received 100 mg/kg licorice extract daily by gavage along with a single-dose injection of 0.2 mg estradiol valerate once a day intramuscularly for 3 wk.

• Group IV: mice received 150 mg/kg licorice extract daily by gavage along with a single-dose injection of 0.2 mg estradiol valerate once a day intramuscularly for 3 wk (Figure 1).

Additionally, in the present study, 0.2 mg/kg body weight (BW) single dose of EV (Aburaihan, Iran) was injected subcutaneously for 21 days (21). Studies have shown that the administration of estradiol valerate leads to anovulation and cystic ovarian morphology (22).

### Preparation of licorice extract

The licorice was collected from a local farm in Urmia, north-west of Iran. The collected samples were identified by the Plant Botanical Laboratory, Faculty of Agriculture Science, Urmia University of Iran. The plant was washed with water and dried in the shade. Then, they were pulverized into a powder using a mixer grinder. To prepare the hydro-alcoholic extract, the powder of the dried licorice was extracted for 72 hr in ethanol 80%, following a solvent evaporation using a rotary evaporator. Next, it was completely dried and collected. Finally, the extract of the licorice was dissolved in normal saline to achieve an appropriate concentration.

### Blood sampling

After the injection, the female mice were sacrificed under anesthesia, before drawing blood (2 mL) from the heart. Blood serum was separated by centrifuging the blood samples at 6000 g for 5 min. Serum samples were stored at -70°C for future analysis. Serum testosterone and estrogen concentrations were assessed through immune-radiometric techniques using commercially available Elisa kits (diaplus, USA) according to the manufacturer's guidelines (23).

### Histological analysis

Ovarian tissue was removed for histological examination following fixation in 10% formalin. Afterward, the samples were cut serially at 5 μm and stained by hematoxylin and eosin. In the final stage, the stained slides taken from the ovaries were observed under a light microscope. The observed follicle was defined in six groups based on the morphology and diameter: primordial follicles (PRIF), in which the oocyte was closely surrounded by one layer of flat granulosa cells; Primary follicles (PF), where the growing oocyte was surrounded by one layer of cuboidal granulosa cells; preantral follicles (PAF), where several layers of granulosa cells surround the oocyte with no cavity within; Antral follicles (AF), where the oocyte is surrounded by cumulus oophorus cells within the cavity of antrum forming; Cystic follicles (CF); and corpus luteum (CL). Thereafter, all follicles were classified as healthy or atretic. The characterization of follicular atresia was done by the presence of pyknotic nuclei in the granulosa cells, degeneration of the granulosa cell layer from the basement membrane, and change in the oocyte morphology. Follicles were then counted in each section and classified according to the work of Radavelli-Bagatini (24, 25).

### Oocyte collection

The NMRI female mice were superovulated by an intraperitoneal injection of 7.5 IU of pregnant mare serum gonadotropin (PMSG, Folligon, Netherlands). After 48 hr, they were euthanized by cervical dislocation, and ovaries were dissected and washed several times before being transferred into a preheated dissecting medium containing α-MEM supplemented with 5% FCS, 100 μg/ml penicillin, and 50 μg/ml streptomycin. under a stereomicroscope Germinal vesicle (GV)-stage oocytes of the ovarian follicles were aseptically harvested by aspiration from follicles with a 26-gauge needle in a sterile petri dish, and released in the culture medium and washed several times (26, 27).

### In vitro maturation (IVM)

The IVM medium consisting of complete MEM containing 100 IU/ml recombinant human follicular-stimulating hormone (rhFSH), and 7.5 IU/ml human chorionic gonadotrophin (hCG). The GV was transferred to 25 µl drops covered with mineral oil and cultured for 16-18 hr at 37°C in 5% CO${}_{2}$ and humified atmosphere. The maturation status of the oocytes, according to polar body extrusion, was assessed under a stereomicroscope (Model TL2, Olympus Co., Tokyo, Japan). Oocytes maturation was evaluated by detecting the first polar body extrusion as an indicator of the oocytes having reached the metaphase II (MII) stage. Oocytes with the extruded first polar body (PB1) were used for IVF (28).

### IVF

The oocytes in the MII stage were obtained from the different groups and fertilized by sperm acquired from the cauda epididymides of 8-10 wk-old male mice with proven fertility. The epididymis was isolated from testes and placed in a petri dish containing 1 ml of equilibrated HTF medium supplemented with 4 mg/ml BSA. The epididymides were then cut and gently pressed allowing the spermatozoa to swim out into the culture media, and then allowed to incubate at 5% CO${}_{2}$ and 37°C for 30 min. Thereafter, spermatozoa were added to the 20 µl IVF droplets containing the oocytes and incubated again at 37°C, 5% CO${}_{2}$ humidified atmosphere for 4-6 hr. Resulting embryos were then cultured at 37°C in 5% CO${}_{2}$ humidified incubator for 5 hr (28).

### Assessment of oocyte maturation

Finally, the number of oocytes at GV, MII stages, and the number of two-pronuclear (2PN) formations were recorded using a stereo microscope. While the two-cell embryos were evaluated 24 hr after fertilization, the percentage of blastocyst-stage embryos was calculated on days 4 and 5 of fertilization (28).

### Ethical consideration

All animal experiments and study protocols were approved by the educational assistant of the Faculty of Science, University of Urmia, Urmia, Iran.

### Statistical analysis

Data presented as mean ± standard deviation (SD). The differences between groups were analyzed by ANOVA, followed by Tukey's test using the SPSS software (Statistical Package for the Social Sciences, version 19. P ≤ 0.05 was deemed statistically significant.

## 3. Results

### Hormonal concentrations

As shown in Table I, testosterone and estrogen levels increased significantly in the PCOS group compared to the control group (p ≤ 0.001) (Table I). The administration of licorice at 100 mg/kg/day and 150 mg/kg/day significantly reduced the testosterone and estrogen levels in the experimental groups compared to the PCOS group after 3 wk (p ≤ 0.001). However, the testosterone and estrogen levels (p = 0.18, p = 0.27) were not significantly different between the experimental groups (Table I).

### Ovarian morphology

**Figure 2:**
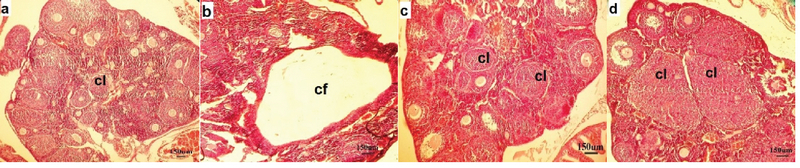
Histological changes in the ovarian tissue. Control (a): Section of ovary from a control group showing primordial follicles (PRIF), primary follicles (PF), preantral follicles (PAF), and antral follicles (AF). PCOS (b): Section of ovary from a PCOS group showing cystic follicle and atretic follicle. (c): Section of ovary from the experimental group (pro100) showing increased corpus luteum. (d): Section of ovary from the experimental group (pro150) showing increased corpus luteum.

In the control group, ovarian tissues had normal histological appearance (Figure 2a). The number of healthy PRIF was not significantly different between the groups. While in the PCOS group, the number of healthy follicles were lower than the control group (PF (p = 0.002), PAF (p = 0.02), AF (p = 0.01)) (Table II). In the experimental groups, the number of healthy follicles PF (p = 0.001, p = 0.002), PAF (p = 0.01) were higher than the PCOS group. Moreover, there were no significant changes in the number of healthy follicles in the experimental groups (Table II). Morphological studies in the PCOS group showed that the number of atretic follicles PRIF (p = 0.01), PF (p = 0.002) in this group were significantly higher compared to the control group (Table III). Also, an evaluation of the number of atretic follicles PF (p ≤ 0.001), PAF (p = 0.02, p = 0.03) showed a significant decrease in the experimental groups compared to the PCOS group (Table III). The PCOS group showed a highly significant increase in CF (p = 0.001) (Figure 2b). And a reduction in the number of corpora luteal (p = 0.02) as compared to the control group (Figure 2c, d). Also, in experimental groups, CF (p = 0.02, p = 0.01) were lower than the PCOS group and an increase in the number of CL (p = 0.001, p ≤ 0.001). However, there were no significant changes in the number of atretic follicles in the experimental groups (Table IV).

### IVM of oocytes

As shown in Table V, the percentage of MII, fertilization rates, and blastocyst decreased significantly in the PCOS group compared to the control group (p ≤ 0.05). The daily oral supplementation of 100 mg/kg/day and 150 mg/kg/day licorice extract for 3 wk resulted in an increased fertilization rates in the experimental groups in comparison with the PCOS group (p ≤ 0.001). The percentage of MII (p = 0.28), fertilization rates (p = 0.39), and blastocyst (p = 0.91) were not statistically significant between the experimental groups (Table V).

**Table 1:** Effects of licorice extract on testosterone and estrogen levels in PCOS-induced mice


**Groups**	**Testosterone**	**Estrogen**
**Group I**	1.60 ± 0.28	8.85 ± 0.21
**Group II**	4.95 ± 0.21${}^{a*}$	14.90 ± 0.14${}^{a*}$
**Group III**	2.10 ± 0.14${}^{b**}$	10.75 ± 0.49${}^{b**}$
**Group IV**	1.60 ± 0.14${}^{b**}$	10.05 ± 0.35${}^{b**}$
Data presented as Mean ± SD. one-way ANOVA test, *, ** Significant difference between the PCOS and other groups at p < 0.05, and p < 0.01, level respectively ${}^{a}$Significant difference compared to the control group		

**Table 2:** Effects of licorice extract on the healthy follicles number in PCOS-induced mice


**Groups**	**Primordial follicles**	**Primary follicles**	**Preantral follicles**	**Antral follicles**
**Group I**	44.66 ± 2.88	14.66 ± 0.57	7.66 ± 2.08	6.33 ± 1.52
**Group II**	47.33 ± 3.78	6.33 ± 1.52${}^{a**}$	2.33 ± 1.${}^{15a*}$	2.33 ± 0.57${}^{a*}$
**Group III**	52.66 ± 4.04	15.66 ± 2.88${}^{b***}$	6.66 ± 0.57	3.33 ± 0.57
**Group IV**	47.33 ± 2.08	14.66 ± 1.52${}^{b**}$	8.33 ± 2.51${}^{b*}$	2.66 ± 1.52${}^{b*}$
Data presented as Mean ± SD. one-way ANOVA test, *, **, *** Significant difference between the PCOS and other groups at p < 0.05, p < 0.01, and p < 0.001 level respectively. ${}^{a}$Significant difference compared to the control group; ${}^{b}$Significant difference compared to the PCOS group				

**Table 3:** Effects of licorice extract on the atretic follicles number in PCOS-induced mice


**Groups**	**Primordial follicles**	**Primary follicles**	**Preantral follicles**	**Antral follicles**
**Group I**	14.66 ± 1.52	37.66 ± 1.52	9.66 ± 2.51	5.33 ± 0.57
**Group II**	22.00 ± 2.64${}^{a*}$	49.66 ± 1.52${}^{a**}$	14.33 ± 1.52	2.66 ± 1.15
**Group III**	23.33 ± 2.51${}^{b**}$	32.66 ± 2.51${}^{b***}$	8.00 ± 1.73${}^{b*}$	4.33 ± 1.52
**Group IV**	21.33 ± 2.08${}^{b*}$	32.33 ± 3.78${}^{b***}$	8.33 ± 2.51${}^{b*}$	2.66 ± 1.52
Data presented as Mean ± SD. one-way ANOVA test, *, **, *** Significant difference between the PCOS and other groups at p < 0.05, p < 0.01, and p < 0.001 level respectively; ${}^{a}$Significant difference compared to the control group; ${}^{b}$Significant difference compared to the PCOS group				

**Table 4:** Effects of licorice extract on the cystic follicle and corpus luteum number in PCOS-induced mice


**Groups**	**Cystic follicles**	**Corpus luteum**
**Group I**	0 ± 0	7.33 ± 2.08
**Group II**	4.33 ± 0.57${}^{a***}$	1.33 ± 0.57${}^{a*}$*
**Group III**	1.66 ± 0.57${}^{b*}$	12.33 ± 2.08${}^{b***}$
**Group IV**	1.33 ± 1.52${}^{b*}$	13 ± 2.64${}^{b***}$
Data presented as Mean ± SD. one-way ANOVA test, ${}^{*}$, **, *** Significant difference between the PCOS and other groups at p < 0.05, p < 0.01, and p < 0.001 level respectively. ${}^{a}$Significant difference compared to the control group; ${}^{b}$Significant difference compared to the PCOS group		

**Table 5:** Effects of licorice extract on the germinal vesicles (GV), metaphase 2 (MII), fertilization rate, 2-cell stage, and blastocysts in PCOS-induced mice


**Groups**	**GV follicles**	**MII oocytes **	**Fertilized oocytes **	**2-cell stage **	**Blastocyst **
**Group I**	50	82.00 ± 2.82	85.50 ± 2.12	78.50 ± 3.53	64.50 ± 3.53
**Group II**	40${}^{a***}$	65.50 ± 3.53${}^{a}$*	56.50 ± 2.12${}^{a***}$	68.00 ± 1.41	41.00 ± 2.82${}^{a**}$
**Group III**	47	76.50 ± 3.53	78.00 ± 2.82${}^{b***}$	71.50 ± 2.12	55.50 ± 2.12
**Group IV**	42${}^{a*}$	78.50 ± 2.12	87.50 ± 0.70${}^{b***}$	78.00 ± 4.24	62.00 ± 4.24
Data presented as Mean ± SD. one-way ANOVA test, ${}^{*}$, **, *** Significant difference between the PCOS and other groups at p < 0.05, p < 0.01, and p < 0.001 level respectively. ${}^{a}$Significant difference compared to the control group; ${}^{b}$Significant difference compared to the PCOS group					

## 4. Discussion

In the present study, the PCOS phenotype was induced with an aim to assess the effects of two different dosage of licorice extract on histology, maturation, and fertilization rates of the ovary in PCOS-induced mice. Examining the testosterone and estrogen levels, we could validate the effect of licorice extract on the hormonal change. The results obtained from the present study showed increased levels of testosterone and estrogen in the PCOS group, as well as a significant decrease in the number of healthy follicles, CL, and increase in atretic follicles. Moreover, the appearance of the cyst in ovary were observed and matched with the previous studies (29). Follicular development and morphology formation in the ovaries were a result of elevated levels of testosterone and estrogen in the PCOS group (30). This refers to hyperandrogenism that leads to the generation of CF, reduction of healthy follicles, and increase in the atretic follicles (31), thereby consequently reducing the progesterone and estrogen levels due to regression of the CL (32). Licorice is a medicinal herb that is used extensively as it is regarded as an important and safe medicine (32). Nearly 500 components have been identified in the licorice root, and glycyrrhizin and several other flavonoids are the major components and most abundant constituents of licorice (32).

Treating overweight women and alleviating metabolic syndrome using licorice were examined in clinical studies (33). In addition, licorice's effect on premenopausal syndrome and menopause syndromes have also been investigated and it showed promising and effective results in alleviating these syndromes (34). Our findings show that the exposure to licorice, both in doses of 100 mg/kg/day and 150 mg/kg/day, significantly decreased the testosterone and estrogen in PCOS-induced mice. In addition, licorice could increase the number of normal follicles and CL and reduce the number of atretic follicles compared to the PCOS group. There were no significant differences in the testosterone, estrogen, number of follicles among the experimental groups. Similar to these results, a study demonstrated that licorice extract improves the adverse effects resulting from hyperandrogenism due to PCOS in female mice and also has a positive effect on fertility (35).

Several studies showed that licorice extract will lead to a reduction in intraovarian androgen concentration when taken orally. Subsequently, this results in decreasing the levels of androgen synthetized from estrogen, causing positive feedback on the LH secretion (36). Catalyzing the biosynthesis of estrogens from androgens is done by aromatase, and deficiency in the activity of this enzyme can be expected to result in increased ovarian androgen production and development of PCOS (37). Armanini and colleague indicated when 17-hydroxysteroid and 17-20-lyase are blocked and the activity of aromatase increases, there is a significant reduction in serum testosterone levels suggesting that licorice could be considered as adjuvant therapy of PCOS (36). A major finding of this research was that licorice positively affected IVM and IVF demonstrating a significantly higher percentage of oocytes reaching MII and blastocyst stages in both experimental groups as compared to the PCOS group. This is in accordance with the results obtained from Esmailii and colleagues, who showed that licorice consumption improved the oocyte maturation and number of follicles. They also studied the beneficial effects of licorice extract on oocyte maturation and infertility due to its phytoestrogen properties (38).

Previous studies have shown oxidative stress to be a prominent pathological feature of PCOS, and women with PCOS have shown a decrease in their total antioxidant status (37). Additionally, antioxidants and reactive oxygen species (ROS) play a physiological role in the reproductive processes including oocyte maturation and fertilization (39). Several previous investigations have revealed that antioxidants can enhance the maturation of oocytes and have a positive effect on embryo development in mice (38). Ju and colleagues have also demonstrated that licorice flavonoids present a powerful antioxidant activity and are capable of scavenging more free radicals (40). Also, licorice is a source of phytoestrogen containing herbal estrogen that is attributed to the presence of isoflavones that are the key components of the plant (41).

Some articles have reported that phytoestrogens decrease the serum level of androgens by increasing the level of sex hormone-binding globulin (SHBG) (42). The isoflavones glabridin and glabrene have estrogen-like activity (12). Also, a study investigated the role of estrogen in modulating sperm during fertilization (43). Sexual development, such as pubertal commencement, impaired estrous cycling, ovarian function, and that functions of the hypothalamus and pituitary can be regulated by isoﬂavones, including formononetin (44). Additionally, a study by Hajirahimkhan and colleague indicated that liquiritigenin is an active ﬂavonoid isoliquiritigenin that may be cyclized of liquiritigenin (34).

Tung and colleague identified that formononetin and isoliquiritigenin are two active components in licorice root that enhance IVF (19). Although Tung and co-workers have reported a not entirely clear relationship between estrogen and fertilization, the two phytoestrogens described here may promote fertilization (45). It is possible that some of the 500 components of licorice extract may act synergistically to stimulate fertilization (45). We observed that two different doses of licorice as an antioxidant has numerous beneficial effects on the hormonal changes and improved ovarian morphology in mice with PCOS. However, different doses of licorice did not differ in the result. Our findings also showed that antioxidants and phytoestrogen activities of licorice might be useful in the promotion of IVM and fertilization. Therefore, we believe that the licorice extract could be useful for managing PCOS in women. Nevertheless, a limitation of this study was the evaluation of oxidative stress markers.

## 5. Conclusion

According to the results of the present study, both doses of licorice may act as a useful treatment for improving PCOS. Our findings support that oral licorice extract intake can cause a reduction in testosterone and estrogen levels, and significantly enhance oocyte maturation, fertilization, and embryo developmental rates in mice. Also, licorice extract can improve ovary morphology in PCOS-induced mice. However, there was no statistically significant difference between the two experimental groups. Since no difference was found between the doses of 100 mg and 150 mg, these conditions will determine the final dose of atorvastatin for each mouse.

##  Conflict of Interest 

The authors declare that there is no conflict of interest.

## References

[1] Li A, Zhang L, Jiang J, Yang N, Liu Y, Cai L, et al. Follicular hyperandrogenism and insulin resistance in polycystic ovary syndrome patients with normal circulating testosterone levels. *J Biomed Res* 2018; 32: 208–214. 10.7555/JBR.32.20170136 PMC6265400 29760297

[2] Escobar-Morreale HF. Polycystic ovary syndrome: definition, aetiology, diagnosis and treatment. *Nat Rev Endocrinol* 2018; 14: 270–284. 10.1038/nrendo.2018.24 29569621

[3] Nelson VL, Legro RS, Strauss JF, McAllister JM. Augmented androgen production is a stable steroidogenic phenotype of propagated theca cells from polycystic ovaries. *Mol Endocrinol* 1999; 13: 946–957. 10.1210/mend.13.6.0311 10379893

[4] Wood JR, Ho CKM, Nelson-Degrave VL, McAllister JM, Strauss JF, et al. The molecular signature of polycystic ovary syndrome (PCOS) theca cells defined by gene expression profiling. *J Reprod Immunol *2004; 63: 51–60. 10.1016/j.jri.2004.01.010 15284005

[5] Caldwell ASL, Middleton LJ, Jimenez M, Desai R, Mcmahon AC, Allan CM, et al. Characterization of reproductive, metabolic, and endocrine features of polycystic ovary syndrome in female hyper androgenic mouse models. *Endocrinology* 2014; 155: 3146–3159. 10.1210/en.2014-1196 24877633

[6] Zhao JZ, Zhou W, Zhang W, Ge HS, Huang XF, Lin JJ. In vitro maturation and fertilization of oocytes from unstimulated ovaries in infertile women with polycystic ovary syndrome. *Fertil Steril* 2009; 91: 2568–2571. 10.1016/j.fertnstert.2008.03.059 18579137

[7] Chang EM, Song HS, Lee DR, Lee WS, Yoon TK. In vitro maturation of human oocytes. its role in infertility treatment and new possibilities. *Clin Exp Reprod Med* 2014; 41: 41–46. 10.5653/cerm.2014.41.2.41 PMC4102689 25045627

[8] Hosseinkhani A, Asadi N, Pasalar M, Zarshenas MM. Traditional persian medicine and management of metabolic dysfunction in polycystic ovary syndrome. *J Tradit Complement Med* 2017; 8: 17–23. 10.1016/j.jtcme.2017.04.006 PMC5755987 29321985

[9] Lee JH, Jo J. Successful treatment with Korean herbal medicine and lifestyle management in an obese woman with polycystic ovarian syndrome. *Integr Med Res* 2017; 6: 325–328. 10.1016/j.imr.2017.06.002 PMC5605367 28951847

[10] Nomura T, Fukai T, Akiyama T. Chemistry of phenolic compounds of licorice (Glycyrrhiza species) and their estrogenic and cytotoxic activities. *Pure Appl Chem *2002; 74: 1199–1206.

[11] Shang H, Cao S, Wang J, Zheng H, Putheti R. Glabridin from Chinese herb licorice inhibits fatigue in mice. *Afr J Tradit Complement Altern Med *2009; 7: 17–23. 10.4314/ajtcam.v7i1.57225 PMC3005384 21304608

[12] Vaya J, Belinky PA, Aviram M. Antioxidant constituents from licorice roots: isolation, structure elucidation and antioxidative capacity toward LDL oxidation. *Free Radic Biol Med* 1997; 23: 302–313. 10.1016/s0891-5849(97)00089-0 9199893

[13] Corson SL. Efficacy and clinical profile of a new oral contraceptive containing norgestimate. US clinical trials. *Acta Obstet Gynecol Scand* 1990; 152 (Supp1.): 25–31. 10.3109/00016349009156503 2189282

[14] Tamura Y, Nishikawa T, Yamada K, Yamamoto M, Kumagai A. Effects of glycyrrhetinic acid and its derivatives on delta 4-5 alpha-and 5 beta-reductase in rat liver. *Arzneimittelforschung* 1979; 29: 647–649. 582760

[15] Latif SA, Conca TJ, Morris DJ. The effects of the licorice derivative, glycyrrhetinic acid, on hepatic 3α-and 3β-hydroxysteroid dehydrogenases and 5α-and 5β-reductase pathways of metabolism of aldosterone in male rats. *Steroids *1990; 55: 52–58. 10.1016/0039-128x(90)90024-6 2326827

[16] Lakshmi T, Geetha RV. Glycyrrhiza glabra linn commonly known as licorice: a therapeutic review. *Int J Pharm Pharm Sci *2011; 3: 20–25.

[17] Sakamoto K, Wakabayashi K. Inhibitory effect of glycyrrhetinic acid on testosterone production in rat gonads. *Endocrinol Jpn* 1988; 35: 333–342. 10.1507/endocrj1954.35.333 2850159

[18] Arentz S, Smith CA, Abbott J, Fahey P, Cheema BS, Bensoussan A. Combined lifestyle and herbal medicine in overweight women with Polycystic Ovary Syndrome (PCOS): A randomized controlled trial. *Phytother Res *2017; 31: 1330–1340. 10.1002/ptr.5858 PMC5599989 28685911

[19] Tung NH, Shoyama Y, Wada M, Tanaka H. Two activators of in vitro fertilization in mice from licorice. *Biochem Biophys Res Commun *2015; 467: 447–450. 10.1016/j.bbrc.2015.09.088 26392313

[20] Yang H, Kim HJ, Pyun BJ, Lee HW. Licorice ethanol extract improves symptoms of polycytic ovary syndrome in Letrozole-induced female rats. *Integr* *Med Res* 2018; 7: 264–270. 10.1016/j.imr.2018.05.003 PMC6160501 30271715

[21] Barzegar MH, Khazali H, Kalantar SM, Khoradmehr A. Effect of Citrullus colocynthis hydro-alcoholic extract on hormonal and folliculogenesis process in estradiol valerate-induced PCOs rats model: An experimental study. *Int J Reprod Biomed* 2017; 15: 661–668. PMC5767647 29387832

[22] Mesbah F, Moslem M, Vojdani Z, Mirkhani H. Does metformin improve in vitro maturation and ultrastructure of oocytes retrieved from estradiol valerate polycystic ovary syndrome-induced rats. *J Ovarian Res *2015; 8: 74–83. 10.1186/s13048-015-0203-x PMC4650318 26577050

[23] Jahan S, Abid A, Khalid S, Afsar T, Ul-Ain Q, Shaheen G, et al. Therapeutic potentials of Quercetin in management of polycystic ovarian syndrome using Letrozole induced rat model: A histological and a biochemical study. *J Ovarian Res *2018; 11: 26–35. 10.1186/s13048-018-0400-5 PMC5883607 29615083

[24] Radavelli-Bagatini S, Blair AR, Proietto J, Spritzer PM, Andrikopoulos S. The New Zealand obese mouse model of obesity insulin resistance and poor breeding performance: evaluation of ovarian structure and function. *J Endocrinol *2011; 209: 307–315. 10.1530/JOE-11-0022 21429962

[25] Ahmadi M, Rostamzadeh A, Fathi F, Mohammadi M, Rezaie MJ. The effect of Melatonin on histological changes of ovary in induced polycystic ovary syndrome model in mice. *Middle East Fertility Society Journal* 2017; 22: 255–259.

[26] Belbasi M, Jorsaraei SGA, Gholamitabar Tabari M, Khanbabaei R. Effect of fetal mouse lung tissue Co-culture on in vitro maturation of mouse immature oocytes. *Cell J *2017; 19: 476–481. 10.22074/cellj.2017.3866 PMC5572296 28836410

[27] Castro SV, Carvalho AA, Silva CMG, Santos FW, Campello CC, de Figueiredo JR, et al. Frozen and fresh ovarian tissue require different culture media to promote in vitro development of bovine preantral follicles. *Biopreserv Biobank *2014; 12: 317–324. 10.1089/bio.2014.0020 25340940

[28] Tavana S, Eimani H, Azarnia M, Shahverdi A, Eftekhari-Yazdi P. Effects of saffron (Crocus sativus L.) aqueous extract on in vitro maturation, fertilization and embryo development of mouse oocytes. *Cell J* 2012; 13: 259–264. PMC3584475 23507933

[29] Manneras L, Cajander S, Lonn M, Stener-Victorin E. Acupuncture and exercise restore adipose tissue expression of sympathetic markers and improve ovarian morphology in rats with dihydrotestosterone-induced PCOS. *Am J Physiol Regul Integr Comp Physiol *2009; 296: 1124–1131. 10.1152/ajpregu.90947.2008 19158405

[30] Dewailly D, Robin G, Peigne M, Decanter C, Pigny P, Catteau-Jonard S. Interactions between androgens, FSH, anti-Mullerian hormone and estradiol during folliculogenesis in the human normal and polycystic ovary. *Hum Reprod Update *2016; 22: 709–724. 10.1093/humupd/dmw027 27566840

[31] De Leo V, la Marca A, Ditto A, Morgante G, Cianci A. Effects of metformin on gonadotropin-induced ovulation in women with polycystic ovary syndrome. *Fertil Steril* 1999; 72: 282–285. 10.1016/s0015-0282(99)00208-3 10438996

[32] Tung NH, Shoyama Y, Wada M, Tanaka H. Improved in vitro fertilization ability of mouse sperm caused by the addition of licorice extract to the preincubation medium. *The Open Reproductive Science Journal *2014; 6: 1–7.

[33] Madak-Erdogan Z, Gong P, Zhao YC, Xu L, Wrobel KU, Hartman JA, et al. Dietary licorice root supplementation reduces diet-induced weight gain, lipid deposition, and hepatic steatosis in ovariectomized mice without stimulating reproductive tissues and mammary gland. *Mol Nutr Food Res* 2016; 60: 369–380. 10.1002/mnfr.201500445 PMC4738101 26555669

[34] Hajirahimkhan A, Simmler C, Yuan Y, Anderson JR, Chen SN, Nikolić D, et al. Evaluation of estrogenicactivity of licorice species in comparison with hops used in botanicals for menopausal symptoms. *PLoS One* 2013; 8: e67947: 1–11. 10.1371/journal.pone.0067947 PMC3709979 23874474

[35] Ahmadi A, Mostafavi M. [Study on the effects of licorice root hydroalcoholiclicorice extract on mice uterus histological structure and level of testosterone improvement with hyperandrogenism following experimental polycystic ovary syndrome]. *The Journal of Urmia University of Medical Sciences* 2015; 26: 571–581. (in Persian)

[36] Armanini D, Castello R, Scaroni C, Bonanni G, Faccini G, Pellati D, et al. Treatment of polycystic ovary syndrome with spironolactone plus licorice. *Eur J Obstet Gynecol Reprod Biol* 2007; 131: 61–67. 10.1016/j.ejogrb.2006.10.013 17113210

[37] Jungbauer A, Medjakovic S. Phytoestrogens and the metabolic syndrome. *J Steroid Biochem Mol Biol *2014; 139: 277–289. 10.1016/j.jsbmb.2012.12.009 23318879

[38] Esmailii Z, Hayati Roodbari N, Mohammady Gorji S, Parivar K. [Effect of licorice plant (Glycyrrhiza glabra) on in vitro maturation of immature oocytes and embryonic development in NMRI mice]. *J Mazandaran Univ Med Sci* 2017; 27: 26–37. (in Persian)

[39] Gupta S, Malhotra N, Sharma D, Chandra A, Ashok A. Oxidative stress and its role in female infertility and assisted reproduction: clinical implications. *International Journal of Fertility and Sterility* 2009; 2: 147–164.

[40] Ju HS, Li XL, Zhao BL, Han ZW, Xin WJ. Effects of glycyrrhiza flavonoid on lipid peroxidation and active oxygen radicals. *Yao Xue Xue Bao *1989; 24: 807–812. 2618676

[41] Somjen D, Knoll E, Vaya J, Stern N, Tamir S. Estrogen-like activity of licorice root constituents: glabridin and glabrene, in vascular tissues in vitro and in vivo. *J Steroid Biochem Mol Biol* 2004; 91: 147–155. 10.1016/j.jsbmb.2004.04.003 15276622

[42] Low YL, Dunning AM, Dowsett M, Folkerd E, Doody D, Taylor J, et al. Phytoestrogen exposure is associated with circulating sex hormone levels in postmenopausal women and interact with ESR1 and NR1I2 gene variants. *Cancer Epidemiol Biomarkers Prev* 2007; 16: 1009–1016. 10.1158/1055-9965.EPI-06-0899 17507630

[43] Bathla H, Guraya SS, Sanghal GK. Role of estradiol in the capacitation and acrosome reaction of hamster epididymal spermatozoa in the isolated uterus of mice incubated in vitro. *Indian J* *Physiol Pharmacol *1999; 43: 211–217. 10365314

[44] Kim SH, Park MJ Effects of phytoestrogen on sexual development. *Korean J* *Pediatr* 2012; 55: 265–271. 10.3345/kjp.2012.55.8.265 PMC3433562 22977438

[45] Tung NH, Tanaka H, Tsujimura A, Miyagawa Y, Wada M, Fujii S, et al. In vitro fertilization with mouse sperm activated by components of licorice root extract. *Nat Prod Chem Res* 2016; 4: 1–4.

